# A Highly Sensitive Refractometric Sensor Based on Cascaded SiN Microring Resonators

**DOI:** 10.3390/s131114601

**Published:** 2013-10-28

**Authors:** Vanessa Zamora, Peter Lützow, Martin Weiland, Daniel Pergande

**Affiliations:** Fraunhofer Institute for Telecommunications, Heinrich Hertz Institute, Einsteinufer 37, Berlin 10587, Germany; E-Mails: peter.luetzow@hhi.fraunhofer.de (P.L.); martin.weiland@hhi.fraunhofer.de (M.W.); daniel.pergande@hhi.fraunhofer.de (D.P.)

**Keywords:** microring resonators, refractometric sensors, Vernier effect, silicon nitride, integrated optical devices

## Abstract

We investigate a highly sensitive optical sensor based on two cascaded microring resonators exploiting the Vernier effect. The architecture consists of two microrings with a slight difference in their free spectral ranges. This allows the generation of the Vernier effect for achieving ultra-high sensitivities. The sensor chip was fabricated using a silicon nitride platform and characterized with isopropanol/ethanol mixtures. A sensitivity of 0.95 nm/% was found for isopropanol concentrations in ethanol ranging from 0% to 10%. Furthermore, a collection of measurements was carried out using aqueous sodium chloride (NaCl) in solutions of different concentrations, confirming a high sensitivity of 10.3 nm/% and a bulk refractive index sensitivity of 6,317 nm/RIU. A limit of detection of 3.16 × 10^−6^ RIU was determined. These preliminary results show the potential features of cascaded silicon nitride microring resonators for real-time and free-label monitoring of biomolecules for a broad range of applications.

## 1. Introduction

The development of integrated optical sensors with high precision and fast response times allows the elaboration of reliable and portable lab-on-a-chip (LOC) optical devices for sensing applications. The sensor elements exhibit remarkable features such as high sensitivity, electromagnetic immunity, the potential for high volume production, as well as hybrid integration with fluidic and electronic microsystems. Most of them are sensitive enough to identify biomolecules without fluorescent markers, thus enabling reduced preparation efforts and costs. The selectivity of such sensors to diverse target molecules by probe surface functionalization has been already demonstrated. Fields of application of LOC-based sensors may encompass the areas of medical research, healthcare, biological recognition, process control including food technology, environment monitoring, as well as safety and security purposes.

In particular, resonance-based optical sensors have been widely studied due to their positive features regarding miniaturization and multiplexing potential. Their work principle is based on the change of the refractive index by capturing analyte molecules. This induces a perturbation in the resonance condition causing a wavelength shift which is monitored as a function of the analyte deposition. Several types of sensor elements have been investigated for this purpose, such as microdisk resonators [1], microring resonators (MRRs) [2–4], and photonic crystals cavities [5]. MRRs have become a favorite structure of resonance-based sensors because of their performance and potential for high volume fabrication. The high quality (*Q*) factor of MRRs results in low limits of detection (LODs) ranging from 10^−4^ refractive index units (RIU) to 10^−6^ RIU [2–4]. In addition, arrays of MRRs for the detection of multiple species at bulk refractive index sensitivities (BRISs) ranging from 190 nm/RIU to 248 nm/RIU were reported using slot waveguide MRRs [6] and a technique based on the frequency modulation for read-out of a sequence of individual MRRs fed by a common bus waveguide [7]. However, the reported sensitivities and LODs are still not sufficient to detect and analyze low concentrations of molecules.

Recently, cascaded microring resonators (CMRRs) exploiting the Vernier effect have been proposed as a highly sensitive optical sensor. This novel architecture was firstly analyzed as a digital sensor [8]. Later, the structure was fabricated on a silicon-on-insulator (SOI) platform and characterized in aqueous medium, reaching a BRIS of 2,169 nm/RIU [9]. This value was improved by two orders of magnitude by using suspended Si-nanowires in this type of architecture [10]. A low-cost intensity interrogation method of CMRRs using a broadband source also was reported [11]. All of them operate in TE mode. In addition, cascaded SOI MRRs were designed and fabricated for operating in TM mode, where a BRIS of 24,300 nm/RIU was demonstrated [12].

In this paper, we present an optical sensor that consists of two cascaded silicon nitride (SiN) MRRs for sensing applications. The theoretical analysis and the fabrication of CMRRs on a SiN platform were reported previously [13,14]. Two types of analyte-isopropanol/ethanol mixtures and sodium chloride (NaCl) in aqueous solutions–were used for the characterization of the sensor in TE mode. Here, real-time measurements were performed resulting in a BRIS of 6,317 nm/RIU for NaCl solutions. Moreover, SiN waveguides can spectrally be tuned via integrated microheater elements due to the thermo-optic dependency of the SiN material. The waveguides have low optical losses over a wide wavelength range. Thereby, sensors based on cascaded SiN MRRs show a high grade of flexibility to operate in different wavelength windows, even at visible wavelengths.

## 2. Sensor Structure

**Figure 1.**
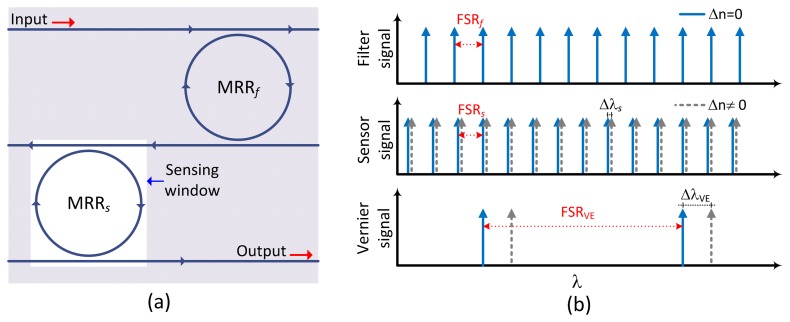
(**a**) Schematic architecture of the optical sensor based on CMRRs. (**b**) Vernier principle employed for sensing applications.

The sensor architecture shown in Figure 1a consists of two cascaded MRRs in an add/drop configuration. The filter element (MRR*_f_*) and the sensor element (MRR*_s_*) are connected via a common bus waveguide, defining the output drop port of the MRR*_f_* as the input port of the MRR*_s_*. A top cladding layer covers the complete structure for the interaction with the analyte sample except the MRR*_s_*. The Vernier operation in two cascaded systems is due to the difference in the free spectral range (*FSR*) as illustrated in Figure 1b, resulting in a large period (*FSR_VE_*) between two consecutive maximum peaks. An index change (Δn ≠ 0) in the sensor element (dashed gray arrows) causes a large wavelength shift (Δλ_VE_) compared to the wavelength shift (Δλ_s_) for the individual sensor element.

For optical interfacing between the sensor chip and the feeding optical fibers, tapered grating couplers have been integrated at the ends of the waveguides for efficient optical interfacing at moderate alignment tolerances. A coupling efficiency of up to 60% in [15] has been reported using grating couplers integrated on SiN waveguides.

The transmission spectrum of CMRRs is the spectral product of the transmitted light by each individual MRR. It consists of a periodicity of resonances peaks whose maximum amplitude occurs by the complete overlap of peaks of the MRR*_f_* and the MRR*_s_*. The resulting transmission peaks have a series of envelopes where each one of them is fitted with a Lorentzian function. The spectral distance (*FSR_VE_*) between two consecutive Vernier peaks at the center of the envelope is defined by [9]:

(1) $${\text{FSR}}_{VE}={\text{FSR}}_{f}\;\cdot \;{\text{FSR}}_{s}/|{\text{FSR}}_{s}-{\text{FSR}}_{f}$$

Here, the *FSR_VE_* is inversely proportional to the difference of individual *FSRs* calculated from *FSR* = *λ^2^*/*n_g_L*, where *n_g_* is the group index of the waveguide and *L* is the perimeter of the MRR.

The sensitivity of the individual MRR*_s_*, which is related to the sensitivity of the waveguide, can be obtained by *S* = *λ*/*n_g,s_*·δ*n_s_*/δ*n_medium_*, where δ*n_s_* is the effective index change and δ*n_medium_* is the index change caused by the analyte amount. According to the Vernier effect, the sensitivity (*S_VE_*) of CMRRs is simply the sensitivity of the sensor element increased by an amplification factor (*M*) [9]:

(2) $$S_{VE}=({\text{FSR}}_{f}/\left|{\text{FSR}}_{s}-{\text{FSR}}_{f}\right|)\;\cdot \;(\lambda /n_{g,s}\;\cdot \;\delta n_{s}/{\delta n}_{\text{medium}})=M\cdot S$$

Note that the factor *M* is directly related to the difference of the individual *FSRs*. In this context, small amounts of analyte on the sensor element surface can be detected via CMRRs fabricated with a small difference in *FSRs*.

## 3. Fabrication

**Figure 2.**
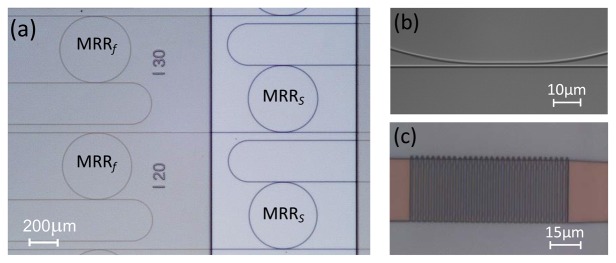
Optical microscope images of the fabricated sensors in SiN material: (**a**) CMRRs covered with a BCB passivation layer and opened on the sensor elements. (**b**) The coupling region between the bus waveguide and the MRR waveguide with a straight section length of 20 μm. (**c**) The tapered grating coupler with period of 1.2 μm used for coupling of the light into the sensor chip.

The sensor was designed on the SiN material platform and fabricated *via* a mask-based UV patterning process followed by an RIE etching process [7]. The ridge SiN waveguides (*n*_SiN_ = 1.99) of 1.25 μm width that form the sensor were etched at a depth of 80 nm, starting from an initial SiN layer of 250 nm thickness deposited on a thermal SiO_x_ layer (*n*_SiOx_ = 1.45) of 8 μm thickness. The designed waveguide only supports the fundamental TE mode at 1,500 nm. The final MRRs had a racetrack shape with a coupler gap (*g*) of 1 μm and different straight section lengths (*l*) between 10 μm and 50 μm. The MRRs had radii of R*_s_* = 199 μm and R*_f_* = 200 μm, respectively. Finally, the complete structure was covered with a 4 μm-thick benzocyclobutene (BCB) layer (*n*_BCB_ = 1.56), which was only opened on MRR*_s_*s by using an extra photolithography step. Figure 2 shows optical microscope images of fabricated CMRR structures. The coupling region of waveguides for *l* = 20 μm and the tapered grating coupler with a period of 1.2 μm, a grating width of 0.6 μm, a spacing of 0.6 μm, and an initial width of 30 μm are also shown in Figures 2b,c, respectively.

Individual *FSRs* were designed at 1,550 nm that correspond to values of *FSR_f_* = 1.006 nm and *FSR_s_* = 0.986 nm, respectively. The MRR*_f_* had a group index of *n*_g,_*_f_* = 1.8215 and a perimeter of *L_f_* = 1310.3 μm. The MRR*_s_* had values of *n*_g,_*_s_* = 1.849 and *L_s_* = 1316.6 μm for operation in water (*n*_medium_ = 1.33). From Equation (1), the theoretical *FSR_VE_* of 49.6 nm was estimated resulting from a difference of *FSRs* of about 0.02 nm. As for the Vernier sensitivity of the sensor, an amplification factor of *M* = 50.3 over the sensitivity of the single sensor element was found from Equation (2).

## 4. Results and Discussion

**Figure 3.**
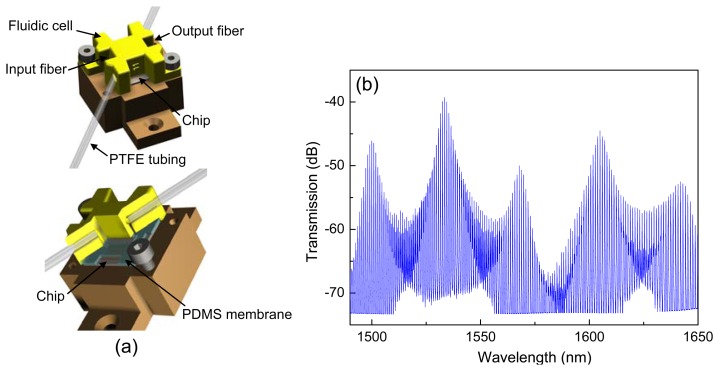
(**a**) Schematic diagram of the complete configuration of sensor chip and fluidic cell. (**b**) Transmission spectrum of the sensor when the sensor element is covered with DI water. Structural parameters: R*_f_* = 199 μm, R*_s_* = 200 μm, *l* = 30 μm and *g* = 1 μm.

To measure the transmission spectrum of the sensor chip, a tunable laser source (TLS)–with an internal piezoelectric element for operation in a dynamic sweep mode or for operation in “digital” wavelength steps of 1 pm–an optical fiber controller for polarization adjustment, and a photodetector were used. The optical interfacing was done by using cleaved optical fibers aligned to tapered grating couplers, which were localized at the ends of the waveguides. A fluidic cell made of acrylate material was fabricated *via* 3D printing technique (ProJet HD 3000 from 3D Systems, Inc., Rock Hill, SC, USA) and was deposited on top of the 10 × 10 mm^2^ sensor chip in order to allow the measurements of different liquid solutions. A PDMS soft membrane was inserted for a better sealing to the sensor chip. The 1/16″ PTFE tubing with an inner diameter of 250 μm was connected to the inlet and the outlet of the fluidic cell, respectively. The solution transport was realized using a syringe pump connected to the end of the outlet tubing. The analyte flowed through inlet tubing with a velocity of 10 μL/min. Figure 3a shows a diagram of the device.

Figure 3b shows the transmission spectrum of the sensor when deionized (DI) water covers the sensing window. Here, five Vernier peaks were measured between 1,490 nm and 1,650 nm. The single Vernier peak, composed by a series of resonance peaks, exhibits an envelope as mentioned above. The analysis of the Vernier peak is based on a Lorentzian fit of the envelope, which is calculated from the maxima of the resonance peaks. The fitted envelopes of two Vernier peaks centered at 1,533.52 nm and 1,568.06 nm showed full-widths at half-maximum (FWHMs) of 4.08 nm and 5.28 nm, respectively. The corresponding *FRS_VE_* was found to be of 34.54 nm. The MRR*_s_* showed experimentally a *Q* factor of 1.8 × 10^4^ and an *FSR_s_* of 0.93 nm, whereas the MRR*_f_* exhibited a *Q* factor of 2.1 × 10^4^ and an *FSR_f_* of 0.95 nm. According to Equation (1), the *FRS_VE_* can be calculated by using individual *FSRs*; the difference of individual *FSRs* was 0.02 nm. Consequently, a theoretical *FRS_VE_* of 44.17 nm was obtained by using the experimental *FSRs*. The difference between the theoretical *FSR_VE_* and the experimental *FSR_VE_* can be related to the waveguide dispersion, which was not taken into account in Equation (1). Note that the amplitude of the Vernier peaks also varies in Figure 3 due to the wavelength dependency on the efficient coupling of the grating couplers.

### 4.1. Isopropanol/Ethanol Measurements

**Figure 4.**
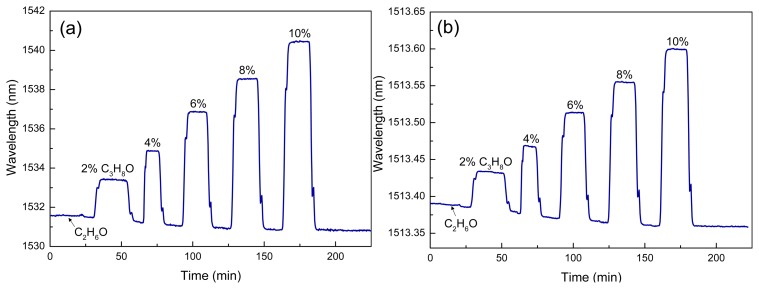
Real-time monitoring of the wavelength shift of isopropanol (C_3_H_8_O)/ethanol (C_2_H_6_O) mixtures for: (**a**) the CMRRs-based sensor and (**b**) the single sensor element.

Firstly, the sensor chip was investigated in terms of its sensitivity with different isopropanol/ethanol mixtures. Figure 4a shows the real-time measurement of the wavelength shift for one Vernier envelope as a function of the isopropanol concentrations in ethanol at room temperature. The isopropanol amount increases in steps of 2% from 0% up to 10%. The next Vernier peak was initially centered at 1,531.59 nm for ethanol solution. As expected, the Vernier peak was displaced to longer wavelengths when the isopropanol amount increases. A wavelength shift of 9.56 nm was demonstrated for the highest isopropanol concentration. From this, a sensitivity of 0.95 nm/% was calculated. Ethanol was drained over the sensor element approximately 20 min between two analyte samples in order to avoid false wavelength shifts. The baseline drift in Figure 4a is mainly due to temperature effects and could be eliminated by employing a temperature reference on chip (for instance an additional MRR).

A study on the sensitivity was performed by tracking a resonance peak of the single MRR_s_. The wavelength shift monitoring of the resonance in Figure 4b is shown for the previous isopropanol/ethanol mixtures. A wavelength resonance shift of 240 pm was found for 10% isopropanol in ethanol. This results into a sensitivity of 0.02 nm/%. The Vernier sensitivity of the sensor can be estimated from Equation (2) and to be compared to the experimental value. An *M* factor of 47.5 was calculated. The resulting *S_VE_* reached 0.95 nm/%, corresponding very well to the experimental value. The temperature effect on the measurement of isopropanol/ethanol mixtures has been observed as well.

### 4.2. Sodium Chloride (NaCl) Measurements

**Figure 5.**
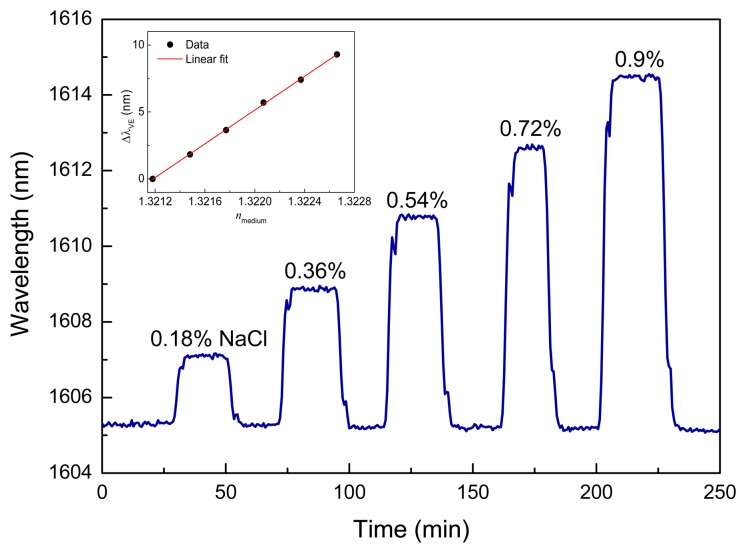
Real-time monitoring of the Vernier envelope for different NaCl solutions in DI water. (Inset) the linear fit (red line) of the wavelength shift *vs.* the refractive index corresponding to the range of NaCl concentrations of 0%–0.9%.

In addition, the sensor chip has been used to measure different NaCl solutions in DI water ranging in concentration from 0% to 0.9% in steps of 0.18%. The corresponding refractive index range was between 1.32118 and 1.32266 with a step of about 3 × 10^−4^ at 25 °C [16]. The wavelength shift of the Vernier envelope centered at 1,605.29 nm was tracked using the respective NaCl solutions, as shown in Figure 5. The NaCl concentration increases up to 0.9%, reaching a maximum wavelength shift of 9.3 nm. After each measurement at a distinct NaCl concentration, the sensor was wiped flowing DI water through the fluidic cell. The sensitivity was of 10.3 nm/%, which is clearly a higher value than the previous one obtained for isopropanol/ethanol mixtures due to the significantly higher refractive index difference between water and NaCl. By using the index change of NaCl solutions, an BRIS of 6,317 nm/RIU was calculated via the slope of the data shown in inset of Figure 5. In contrast to isopropanol/ethanol measurements, the influence of the temperature to the sensor chip was much lower for aqueous solutions. This effect can be caused by the low boiling point of alcohol solutions to temperature changes.

The LOD has been defined as the ratio of the minimum detectable wavelength shift of our system and the sensitivity of the sensor chip. For the minimum detectable wavelength shift, a value of 0.02 nm was found taking into account the noise level of our system as described in [17]. This results in a LOD of 3.16 × 10^−6^ RIU for the proposed sensor.

Finally, Table 1 gives an overview of sensing data for different cascaded optical structures. The Vernier principle has been demonstrated in structures such as MRRs, Mach-Zenhder interferometers (MZIs), and capillary optofluidic rings (OFRRs). All of them show sensitivities up to three orders of magnitude higher than the sensitivity for a single structure. Integrated MZIs are architectures based on a sensing arm and a reference arm connected to Y-junctions for the optical interrogation. The high efficiency of MZIs using an intensity interrogation method is related to the length of their arms. Cascaded MZIs with arms of about millimeters in length become less appropriate for the complete integration of the sensor. However, a single MZI presents high sensitivities and low LODs. Concerning the OFRRs, an effort is being widely performed for their compatibility with integrated waveguide systems. The Vernier operation in this type of structures is achieved by using a reference active fiber and an OFRR. For sensing operation, the modal interaction of the OFRR with the analyte requires a wall thickness of about 1 μm, which is too fragile for practical handling. The advantages of OFRRs are such as a high *Q* factor and their natural fluidic channel that avoids the microfluidic cell integration as in the case of LOC optical devices. Finally, focusing on CMRRs, all of them have been fabricated on SOI platforms and optically characterized in aqueous medium. SOI material permits a CMOS compatible process, which reduces the cost per chip. High sensitivities have been successfully demonstrated using cascaded SOI MRRs. However, the reported LODs are still insufficient compared to, e.g., LODs between 10^−5^ RIU and 10^−8^ RIU obtained via superficial plasmon resonance (SPR) sensors [21,22]. LODs in CMRRs can be improved decreasing the difference of individual *FSRs* [8], consequently, CMRRs should be performed on a technique with low fabrication tolerances as, e.g., the e-beam direct writing process. However, they are still structures in stage of development and improvement.

We conclude by proposing cascaded SiN MRRs as sensor structures equal to SOI MRRs for sensitive biochemical sensing, due to their overall performance in real-time monitoring and sensitivity, e.g., for alcohol and aqueous solutions. SiN MRRs sensors operate over a wide wavelength range [23] and also can be moderately tuned by using integrated microheater elements [7], *i.e.*, the Vernier peak can be centered at any required SiN MRRs wavelength.

## 5. Conclusions

An optical sensor chip based on two cascaded SiN MRRs was experimentally investigated. The sensor was characterized with two types of analyte: isopropanol/ethanol mixtures and aqueous NaCl solutions. A sensitivity of 0.95 nm/% was found for isopropanol/ethanol mixtures compared to the sensitivity of 0.02 nm/% for a single sensor element. Here, an amplification factor of *M* = 47.5 was reported for the actual sensor. Furthermore, the sensing operation was carried out in aqueous solutions of NaCl concentration resulting in a sensitivity of 10.3 nm/% and a BRIS of 6,317 nm/RIU, respectively. In addition, an LOD of 3.16 × 10^−6^ RIU was demonstrated for the proposed sensor. CMRRs based on SiN material are promising candidates for highly sensitive optical sensors at real-time monitoring of biomolecules in wide wavelength range.

## Figures and Tables

**Table 1.** Cascaded optical structures based on the Vernier effect for sensing applications.

**Cascaded Structures**	**Analyte**	***S****_VE_***(nm/RIU)**	**LOD (RIU)**	**Ref.**
SOI MRRs	NaCl	2,169	8.3 × 10^−6^	[9]
	Ethanol	1,300	2.2 × 10^−4^	[18]
SOI MRRs *	NaCl	24,300	4.8 × 10^−6^	[12]
SOI MRRs **	Salt	460,000		[10]
SiN MZIs	NaCl	14,500 (2π)/RIU	1.08 × 10^−6^	[19]
SiO2 OFRRs	Glucose	2,510	1.6 × 10^−5^	[20]
SiN MRRs	NaCl	6,317	3.16 × 10^−6^	This paper

* The fabricated structure operating in TM mode. All of them operate in TE mode.

** CMRRs with a suspended Si-nanowire.
